# Strangulation of the Small Intestine by the Mesentery of the Ileum

**Published:** 1880-10

**Authors:** A. M. Barker


### STRANGULATION OF THE SMALL INTESTINE BY THE MESENTERY OF THE ILEUM.

BY A. M. BARKER, M. D.

Wm. E., a strong healthy looking man, age 25, single, occupation a teamster, was admitted to the Hospital of the Sisters of Charity, about 11 P. M., Aug. 31st. The history of the case previous to his entrance is furnished by Dr. W. H. Slacer.

"I was called at 5 P. M., Aug. 31st, to attend Wm. E. He had been eating very freely of green apples the day previous. He was attacked suddenly about two o'clock on the morning of the 31st with pains in the abdominal region. Was able to be around all day, but suffered considerable pain. Said he had a slight passage from the bowels during the night and voided urine at the time. I found him in pain, pulse 160, feeble, a cold sweat over the entire surface of the body and an urgent desire to urinate, which he was unable to do. The abdomen was hard and dull on percussion; bladder slightly distended. An attempt was made to pass a catheter, but was compelled to relinquish it on account of the patient's resistance and unfavorable surroundings. ¼ grain of morphia sulph. was administered hypodermically, and the patient removed at once to the Hospital."

I saw him for the first time about ten o'clock the next morning. He was in a condition of collapse. The skin was cold and covered with a clammy prespiration; pulse 160, weak and feeble; temperature 102.

He had passed urine quite freely very soon after his admission to the Hospital, and also had a passage from the bowels. The abdomen was very hard, somewhat retracted, not in the least tender, and dull on percussion. He was free from pain and insisted that he was not very sick and that he would be about by the next morning.

Stimulants were administered very freely and morphia sulph. was ordered should pain recur. From his condition I did not think he could possibly live more than four or five hours. At my visit next morning I found him still alive and in much the same condition as upon my first visit, with the exception that he was exceedingly thirsty and had drunk a large quantity of water during the night. He was regurgitating a yellowish looking fluid, free from odor, and with no consistence.

He was still hopeful but very much weaker. The abdomen was not tympanitic, nor swollen, but very hard upon pressure. He died at six o'clock on the evening of Sept. 2d. As he was being carried from the ward of the Hospital, several quarts of yellowish fluid, free from odor, escaped from the mouth.

Autopsy 24 hours after death by Dr. Slacer and myself. We found the abdomen tympanitic and very much swollen.

An incision was made through the abdominal wall and a large amount of bloody serum escaped. The abdominal cavity was found partially filled with this fluid. The intestines were very much distended with gas, and a portion of the smaller were adherent from a recent peritonitis, very red, intensely congested, but not in the least softened. About 3 feet from the ileo-cæcal valve we found that some 4 or 5 feet of the small intestine had passed through a slit in the mesentery of the ileum, which was firmly constricted. Beyond the constriction, the intestine was tightly twisted and tied in a *double knot*, which it was almost impossible to untie. Upon opening the gut it was found free from fæcal matter, but completely filled with what appeared to be pure blood. The amount was estimated to be at least 32 ounces.

The large and small intestines not involved in the constriction were in a healthy condition and not in the least congested. No examination of the heart and lungs was made.

Upon reviewing the case, from the intense thirst, and the large amount of blood found in the intestines and abdominal cavity, from the condition of the intestines and peritoneum we were inclined to believe the patient died from exhaustion caused by the hemorrhage into the bowels and peritoneal cavity. Regarding the correct diagnosis of such cases, I believe it impossible to make one, other than obstruction of the bowels. Regarding the treatment, the only possible chance for recovery would be a resort to laparotomy. The utter impracticability of such an operation in the case reported, was evident at the autopsy, which showed it would have been an impossibility to restore the bowel had abdominal section been attempted.

For a history of similar cases I would refer to the chapters on constrictions, occlusions and displacements of the intestines, by Leichtenstern, in Vol. VII of "Ziemssen's Cyclopœdia of the Practice of Medicine."

